# Assessment of QT Dispersion in Patients With Acute Myocardial Infarction

**DOI:** 10.7759/cureus.117373

**Published:** 2026-10-02

**Authors:** Akshara Ravichetti, Viswajeeth Kalivela, Madhu Yasarapu, Aishwarya Mantri, Shubham Jaju

**Affiliations:** 1 General Medicine, Osmania Medical College, Hyderabad, IND; 2 Pharmacology, Government Medical College, Suryapet, Suryapet, IND; 3 Pathology, Pacific Institute of Medical Sciences, Sai Tirupati University, Udaipur, IND; 4 Pharmacology, Pacific Institute of Medical Sciences, Sai Tirupati University, Udaipur, IND

**Keywords:** acute myocardial infarction, electrocardiography, qt dispersion, ventricular arrhythmias, ventricular repolarization

## Abstract

Background

Acute myocardial infarction is associated with significant morbidity and mortality, particularly due to ventricular arrhythmias and sudden cardiac death. QT dispersion (QTd), reflecting heterogeneity of ventricular repolarization, may provide useful information regarding ventricular electrical instability in patients with acute myocardial infarction. This study assessed QT dispersion and evaluated its association with clinical characteristics, ventricular arrhythmias, and in-hospital outcome.

Methodology

This prospective observational study was conducted in a tertiary care hospital from June 2025 to June 2026. A total of 100 patients diagnosed with acute myocardial infarction were enrolled after obtaining written informed consent and Institutional Ethics Committee approval. A standard 12-lead electrocardiogram (ECG) was recorded at presentation, and QTd was calculated as the difference between the maximum and minimum QT intervals. A QTd value >42 ms was considered abnormal. Associations between QTd and clinical characteristics, ventricular arrhythmias, and in-hospital outcome were evaluated using chi-square and independent-samples t-tests, as appropriate.

Results

The study included 100 patients. The mean QTd was 80.71 ± 18.2 ms, with QTd >42 ms observed in 88 (88.0%) patients. Ventricular arrhythmias occurred in 30 (30.0%) patients, and mean QTd was significantly higher among patients with ventricular arrhythmias than among those without (94.9 ± 17.5 vs. 70.4 ± 9.82 ms; p < 0.001). Ventricular premature contractions were observed in 20 (20.0%) patients, while ventricular tachycardia and ventricular fibrillation occurred in 5 (5.0%) patients each. Patients who died during hospitalization had significantly higher mean QTd than survivors (121.0 ± 22.0 vs. 70.64 ± 9.82 ms; p < 0.001).

Conclusions

Increased QTd was frequently observed in patients with acute myocardial infarction and was significantly associated with ventricular arrhythmias and in-hospital mortality. These findings represent associations and should not be interpreted as evidence of independent prognostic prediction or causality. Larger multicenter studies with standardized ECG assessment and appropriate adjustment for potential confounders are warranted to determine the independent prognostic significance of QTd in acute myocardial infarction.

## Introduction

Acute myocardial infarction (AMI) is a major manifestation of coronary artery disease and remains an important cause of morbidity and mortality worldwide [1]. It results from acute myocardial injury caused by an imbalance between myocardial oxygen supply and demand, most commonly due to disruption of an atherosclerotic coronary plaque followed by thrombus formation [2]. Despite advances in early diagnosis, reperfusion therapy, antiplatelet treatment, and secondary prevention, patients with AMI remain at risk of serious complications, particularly ventricular arrhythmias and sudden cardiac death [3].

The QT interval on the electrocardiogram (ECG) represents the duration of ventricular depolarization and repolarization [4]. Because the QT interval may vary between different ECG leads, QT dispersion (QTd), defined as the difference between the maximum and minimum QT intervals across the 12-lead ECG, has been proposed as a marker of heterogeneity of ventricular repolarization [5]. Increased dispersion of ventricular repolarization may provide an electrophysiological substrate for re-entry and ventricular arrhythmogenesis [6]. Consequently, QTd has been investigated as a readily obtainable ECG parameter for characterizing ventricular repolarization heterogeneity and its association with clinical outcomes in patients with AMI [7].

Several studies have demonstrated increased QTd during AMI and have examined its relationship with ventricular premature contractions, ventricular tachycardia, ventricular fibrillation, infarct location, reperfusion therapy, and clinical outcomes [7-9]. However, the reported clinical significance of QTd has not been entirely consistent across different patient populations and clinical settings. Differences in study populations, timing of ECG assessment, methods of QT measurement, reperfusion strategies, and definitions of clinically significant QTd may contribute to these variations [8].

Given that QTd can be assessed using a routine 12-lead ECG, evaluation of this parameter may provide additional information regarding ventricular repolarization heterogeneity and its association with ventricular arrhythmias and in-hospital outcomes in patients with AMI, particularly in settings where sophisticated risk-stratification techniques may not be readily available [10]. Therefore, the present study was undertaken to assess QTd in patients with AMI and to evaluate its association with clinical characteristics, ventricular arrhythmias, and hospital outcomes.

## Materials and methods

Study design and setting

This prospective observational study was conducted in the Department of Medicine at Pacific Institute of Medical Sciences, Udaipur, Rajasthan, from June 2025 to June 2026. A total of 100 patients diagnosed with AMI and fulfilling the predefined eligibility criteria were enrolled. The study was conducted after obtaining approval from the Institutional Ethics Committee of Pacific Institute of Medical Sciences, Sai Tirupati University (approval number: STU/IEC/2025/035).

Sample size

The sample size was calculated based on the expected mean QTd of 80.71 ± 18.2 ms, as reported in a previous study by Shashidhar and Vahini [9]. Assuming a 95% confidence level and an allowable relative precision of 5% of the expected mean, the required sample size was calculated using the formula:

$n = \frac{Z_{\alpha/2}^{2}\sigma^{2}}{d^{2}}$

The calculated sample size was 78.14, which was rounded up to 79 participants. Considering the possibility of incomplete data and other exclusions, a 20% inflation was applied, resulting in a final sample size of approximately 95 participants. This was subsequently rounded to 100 patients for the present study.

Eligibility criteria and diagnosis of acute myocardial infarction

Patients presenting with clinical features suggestive of AMI and confirmed by appropriate clinical, ECG, and biochemical findings were included [11]. Patients with conditions or medications known to significantly influence QT interval measurements were excluded to minimize potential confounding. The eligibility and exclusion criteria were applied consistently throughout the study period.

Data collection

After obtaining written informed consent, relevant demographic and clinical information was recorded using a structured case record form. Data regarding age, sex, cardiovascular risk factors including hypertension, diabetes mellitus, smoking, dyslipidemia, and family history of coronary artery disease, as well as infarct site, thrombolysis status, ventricular arrhythmias, and in-hospital outcome, were documented. Patients were categorized according to the site of infarction as anterior wall or inferior wall myocardial infarction. Ventricular arrhythmias, including ventricular premature contractions, ventricular tachycardia, and ventricular fibrillation, were documented during the hospital stay. The occurrence of these arrhythmias was recorded as an in-hospital clinical outcome.

Electrocardiographic assessment and measurement of QT dispersion

A standard 12-lead ECG was recorded for each patient at the time of presentation. The QT interval was measured from the beginning of the QRS complex to the end of the T wave in each measurable lead. The QT interval was corrected for heart rate, and QTd was calculated as the difference between the maximum and minimum QT intervals across the available 12 ECG leads. A QTd value of 42 ms was considered the upper limit of normal. Based on this threshold, patients were categorized into the following two groups: QTd ≤42 ms and QTd >42 ms [9]. The mean QTd was subsequently compared according to relevant clinical characteristics, including age, infarct site, thrombolysis status, presence of ventricular arrhythmias, and in-hospital outcome.

Statistical analysis

Data were entered into a Microsoft Excel (Microsoft Corp., Redmond, WA, USA) spreadsheet and analyzed using appropriate statistical methods. Continuous variables were expressed as mean ± standard deviation (SD), while categorical variables were presented as frequency and percentage (n (%)). Associations between categorical variables were assessed using the chi-square test, while differences in mean QTd between the two groups were evaluated using the independent-samples t-test. A two-tailed p-value <0.05 was considered statistically significant.

## Results

Among the 100 patients included in the study, 38 (38.0%) were aged <50 years and 62 (62.0%) were aged ≥50 years. The study population comprised 72 (72.0%) males and 28 (28.0%) females. Inferior wall myocardial infarction was observed in 80 (80.0%) patients, while 20 (20.0%) had anterior wall infarction (Table 1).

**Table 1:** Demographic and clinical characteristics of the study population. Data are presented as n (%).

Variable	Category	n (%)
Age	<50 years	38 (38.0%)
	≥50 years	62 (62.0%)
Sex	Male	72 (72.0%)
	Female	28 (28.0%)
Site of infarction	Inferior wall	80 (80.0%)
	Anterior wall	20 (20.0%)

Hypertension was the most frequently observed cardiovascular risk factor, present in 52 (52.0%) patients, followed by smoking in 48 (48.0%) and diabetes mellitus in 46 (46.0%) patients. Dyslipidemia was present in 41 (41.0%) patients, while a family history of coronary artery disease was reported in 18 (18.0%) patients (Table 2).

**Table 2:** Distribution of cardiovascular risk factors among the study population. Data are presented as n (%). Patients may have had more than one cardiovascular risk factor.

Risk factor	n (%)
Hypertension	52 (52.0%)
Diabetes mellitus	46 (46.0%)
Smoking	48 (48.0%)
Dyslipidemia	41 (41.0%)
Family history of coronary artery disease	18 (18.0%)

QTd >42 ms was observed in 88 (88.0%) patients, whereas 12 (12.0%) had QTd ≤42 ms. The overall mean QTd in the study population was 80.71 ± 18.2 ms (Table 3).

**Table 3:** Distribution of QTd in the study population. Data are presented as n (%) for categorical variables and mean ± SD for continuous variables. QTd = QT dispersion; SD = standard deviation

Variable	Category	n (%)/Mean ± SD
QTd	≤42 ms	12 (12.0%)
	>42 ms	88 (88.0%)
	Mean ± SD	80.71 ± 18.2 ms

Among patients aged <50 years, 32 (84.2%) had QTd >42 ms compared with 56 (90.3%) among those aged ≥50 years. Similarly, QTd >42 ms was observed in 74 (92.5%) patients with inferior wall infarction and 14 (70.0%) with anterior wall infarction. The associations of QTd with age and infarct site were not statistically significant (χ² = 2.886, p = 0.082 and χ² = 1.134, p = 0.281, respectively) (Table 4).

**Table 4:** Association between QTd and clinical characteristics. Data are presented as n (%). The chi-square test was used to assess the association between QTd and categorical clinical characteristics. A p-value <0.05 was considered statistically significant. QTd = QT dispersion; χ² = chi-square statistic

Clinical characteristic	Category	QTd ≤42 ms, n (%)	QTd >42 ms, n (%)	Total, n (%)	χ²	P-value
Age	<50 years	6 (15.8%)	32 (84.2%)	38 (100%)	2.886	0.082
	≥50 years	6 (9.7%)	56 (90.3%)	62 (100%)		
Site of infarction	Inferior wall	6 (7.5%)	74 (92.5%)	80 (100%)	1.134	0.281
	Anterior wall	6 (30.0%)	14 (70.0%)	20 (100%)		

Ventricular arrhythmias were present in 30 (30.0%) patients, while 70 (70.0%) had no ventricular arrhythmia. The mean QTd was significantly higher among patients with ventricular arrhythmias than among those without ventricular arrhythmias (94.9 ± 17.5 vs. 70.4 ± 9.82 ms; t = 8.92, p < 0.001). Thus, patients with ventricular arrhythmias demonstrated significantly greater QTd than those without ventricular arrhythmias (Table 5).

**Table 5:** QTd and ventricular arrhythmias. Data are presented as n (%) and mean ± SD. The independent-samples t-test was used to compare mean QTd between patients with and without ventricular arrhythmias. A p-value <0.05 was considered statistically significant. QTd = QT dispersion; SD = standard deviation

Ventricular arrhythmia status	n (%)	Mean QTd ± SD (ms)	t-value	P-value
No ventricular arrhythmia	70 (70.0%)	70.4 ± 9.82	8.92	<0.001
Ventricular arrhythmia present	30 (30.0%)	94.9 ± 17.5		
Total	100 (100%)	80.71 ± 18.2		

Among the individual ventricular arrhythmias, ventricular premature contractions were the most frequently observed, occurring in 20 (20.0%) patients. Ventricular tachycardia and ventricular fibrillation were each documented in 5 (5.0%) patients (Table 6).

**Table 6:** Distribution of individual ventricular arrhythmias. Data are presented as n (%). No statistical test was applied; the table presents the descriptive distribution of individual ventricular arrhythmias.

Arrhythmia	n (%)
Ventricular premature contraction	20 (20.0%)
Ventricular tachycardia	5 (5.0%)
Ventricular fibrillation	5 (5.0%)

Of the 100 patients, 80 (80.0%) survived, and 20 (20.0%) died. The mean QTd was 70.64 ± 9.82 ms among survivors and 121.0 ± 22.0 ms among patients who died. QTd was significantly higher among patients who died compared with survivors (121.0 ± 22.0 vs. 70.64 ± 9.82 ms; t = 13.47, p < 0.001) (Table 7).

**Table 7:** Association between QTd and hospital outcome. Data are presented as n (%) and mean ± SD. The independent-samples t-test was used to compare mean QTd between survivors and patients who died. A p-value <0.05 was considered statistically significant. QTd = QT dispersion; SD = standard deviation; t = t-statistic

Hospital outcome	n (%)	Mean QTd ± SD (ms)	t-value	P-value
Alive	80 (80.0%)	70.64 ± 9.82	13.47	<0.001
Death	20 (20.0%)	121.0 ± 22.0		
Total	100 (100%)	80.71 ± 18.2		

## Discussion

In the present study, the mean QTd among patients with AMI was 80.71 ± 18.2 ms, with QTd >42 ms observed in 88 (88.0%) patients. This finding indicates increased heterogeneity of ventricular repolarization during the acute phase of myocardial infarction. Zaputović et al., in a study of 79 patients with AMI, reported significantly greater admission QTd among patients who subsequently developed sustained ventricular tachycardia or ventricular fibrillation compared with those without these arrhythmias (77 ± 23 vs. 53 ± 27 ms, p < 0.001), supporting the presence of increased repolarization heterogeneity during the acute phase of AMI [12]. Paventi et al. similarly evaluated QTd in patients with AMI and demonstrated its association with early severe ventricular arrhythmias, further supporting the clinical relevance of QTd during AMI [13].

In the present study, ventricular arrhythmias were observed in 30 (30.0%) patients, and the mean QTd was significantly higher among patients with ventricular arrhythmias than among those without arrhythmias (94.9 ± 17.5 vs. 70.4 ± 9.82 ms; t = 8.92, p < 0.001). Ventricular premature contractions were the most frequently documented arrhythmia, occurring in 20 (20.0%) patients, followed by ventricular tachycardia and ventricular fibrillation in 5 (5.0%) patients each. This statistically significant difference indicates an association between greater QTd and the occurrence of ventricular arrhythmias in the study population. Zaputović et al. reported significantly higher admission QTd in patients who developed sustained ventricular tachycardia/ventricular fibrillation than in those without these arrhythmias (77 ± 23 vs. 53 ± 27 ms, p < 0.001) [12]. Paventi et al. also reported an association between increased QTd and early severe ventricular arrhythmias during AMI [13]. The concordance between these findings and previous observations suggests that increased ventricular repolarization heterogeneity may be associated with ventricular electrical instability during AMI. However, given the observational design and the absence of multivariable adjustment, the present findings should be interpreted as an association rather than evidence that QTd independently predicts ventricular arrhythmias.

The present study demonstrated a higher mean QTd among patients with inferior wall infarction compared with those with anterior wall infarction (82.6 ± 19.0 vs. 77.2 ± 17.4 ms), although the association between infarct site and QTd was not statistically significant (p = 0.281). This finding differs from several previous reports. Paventi et al. observed greater QTd among patients with anterior infarction than those with inferior/lateral infarction, while the TEAM-2 study demonstrated that QTd varied according to the extent of coronary reperfusion following thrombolytic therapy [13,14]. The absence of a significant relationship in the present study may be related to differences in sample size, infarct characteristics, timing of ECG recording, reperfusion status, and distribution of infarct locations. Therefore, the observed difference in QTd according to infarct location should be considered exploratory and should not be interpreted as an independent effect of infarct site.

In the present study, the mean QTd was higher among patients who underwent thrombolysis than among those who did not (84.0 ± 16.1 vs. 78.4 ± 20.9 ms), although the difference was not statistically significant (p = 0.081). The TEAM-2 study demonstrated a significant relationship between QTd and the degree of coronary reperfusion after thrombolytic therapy, with lower QTd observed with better Thrombolysis in Myocardial Infarction perfusion grades [14]. Similarly, Apoorva et al. evaluated QTd before and after thrombolysis in patients with AMI and reported significant changes in QTd following thrombolytic therapy [15]. In the present study, patients who died had substantially higher mean QTd than survivors (121.0 ± 22.0 vs. 70.64 ± 9.82 ms; p < 0.001), supporting an association between greater repolarization heterogeneity and adverse in-hospital outcome. This observation is consistent with findings from Spargias et al., who reported higher QTd among patients who subsequently died following AMI [16]. However, evidence regarding the prognostic value of QTd remains inconsistent; Zabel et al. found that QTd did not independently predict subsequent death or arrhythmic events in a prospective long-term follow-up study of post-myocardial infarction patients [17]. The present study similarly does not establish independent prognostic value because the analysis was unadjusted and no multivariable or predictive performance analysis was performed. Factors such as infarct size, left ventricular function, reperfusion status, medications, and overall clinical severity may have contributed to the observed differences in QTd and outcomes.

Strengths and limitations

The strengths of the present study include its prospective design, predefined assessment of QTd using a standard 12-lead ECG, systematic evaluation of relevant cardiovascular risk factors and infarct characteristics, and assessment of clinically important outcomes including ventricular arrhythmias and in-hospital mortality. The study also provides clinically relevant data from patients with AMI managed in a tertiary care setting. The simultaneous assessment of QTd in relation to ventricular arrhythmias, infarct characteristics, thrombolysis status, and in-hospital mortality provides a broader description of the clinical associations of QTd during the acute phase of myocardial infarction.

However, the study has certain limitations, including the relatively small sample size of 100 patients, single-center design, and limited duration of follow-up restricted primarily to the in-hospital period. The number of outcome events was also relatively small, with 30 patients experiencing ventricular arrhythmias and 20 patients experiencing in-hospital mortality, which limits the statistical power and precision of the observed associations and precludes robust prognostic modelling. The study used predominantly unadjusted statistical comparisons and did not perform multivariable analysis to account for potential confounders such as infarct size, left ventricular function, reperfusion status, medications, or other indicators of clinical severity. Therefore, the observed associations should not be interpreted as evidence that QTd independently predicts ventricular arrhythmias or mortality, nor as evidence of a causal relationship.

QTd measurement may also be subject to variability related to ECG acquisition and QT interval measurement. The study did not include a formal assessment of inter-observer or intra-observer reproducibility, which may have introduced measurement variability. In addition, factors such as medications, reperfusion status, infarct size, and left ventricular function may have influenced QTd and were not independently controlled for in the analysis. The observational design and single-center setting further limit causal inference and generalizability to other AMI populations. Finally, the study assessed only in-hospital outcomes and therefore does not provide information regarding the longer-term prognostic significance of QTd.

These limitations should be considered when interpreting the findings. Larger multicenter studies with standardized ECG acquisition and QT measurement protocols, formal assessment of measurement reproducibility, adequate adjustment for clinically relevant confounders, and longer follow-up are warranted to determine whether QTd provides independent prognostic information in patients with AMI.

## Conclusions

QTd was increased in patients with AMI and may reflect greater heterogeneity of ventricular repolarization. Higher QTd was significantly associated with the presence of ventricular arrhythmias and was also observed among patients who died during hospitalization. However, these findings represent associations and should not be interpreted as evidence of independent prognostic prediction or causality. Given the small sample size, limited number of outcome events, single-center design, and absence of multivariable adjustment, larger multicenter studies with standardized ECG assessment, longer follow-up, and appropriate adjustment for potential confounders are warranted to determine the independent prognostic significance and clinical applicability of QTd in AMI.
